# Human Malignant Melanoma-Derived Progestagen-Associated Endometrial Protein Immunosuppresses T Lymphocytes *In Vitro*

**DOI:** 10.1371/journal.pone.0119038

**Published:** 2015-03-18

**Authors:** Suping Ren, Lina Chai, Chunyan Wang, Changlan Li, Qiquan Ren, Lihua Yang, Fumei Wang, Zhixin Qiao, Weijing Li, Min He, Adam I. Riker, Ying Han, Qun Yu

**Affiliations:** 1 Department of Immunohematology, Beijing Institute of Transfusion Medicine, Beijing, China; 2 Department of poisoning treatment, Beijing 307 hospital, Beijing, China; 3 People’s Hospital of Anqiu City, Anqiu, Shandong, China; 4 Advocate Christ Medical Center, Advocate Cancer Institute, Oak Lawn, IL, United States of America; University Paris Sud, FRANCE

## Abstract

Progestagen-associated endometrial protein (PAEP) is a glycoprotein of the lipocalin family that acts as a negative regulator of T cell receptor-mediated activation. However, the function of tumor-derived PAEP on the human immune system in the tumor microenvironment is unknown. PAEP is highly expressed in intermediate and thick primary melanomas (Breslow’s 2.5mm or greater) and metastatic melanomas, correlating with its expression in daughter cell lines established *in vitro*. The current study investigates the role of melanoma cell-secreted PAEP protein in regulating T cell function. Upon the enrichment of CD3^+^, CD4^+^ and CD8^+^ T cells from human peripheral blood mononuclear cells, each subset was then mixed with either melanoma-derived PAEP protein or PAEP-poor supernatant of gene-silenced tumor cells. IL-2 and IFN-γ secretion of CD4^+^ T cells significantly decreased with the addition of PAEP-rich supernatant. And the addition of PAEP-positive cell supernatant to activated lymphocytes significantly inhibited lymphocyte proliferation and cytotoxic T cell activity, while increasing lymphocyte apoptosis. Our result suggests that melanoma cell-secreted PAEP protein immunosuppresses the activation, proliferation and cytotoxicity of T lymphocytes, which might partially explain the mechanism of immune tolerance induced by melanoma cells within the tumor microenvironment.

## Introduction

Melanoma is one of the most lethal tumors of the skin, responsible for up to 80% of skin cancer deaths [1, 2]. Despite recent exciting advances in the treatment of metastatic melanoma with several newly developed targeted therapies, the majority of patients will still die of their disease. Therefore, continued in-depth analysis of the pathogenesis of melanoma is very important in order to identify more effective treatment options. Recently, we reported that the oncogenesis and development of melanoma are closely related to the expression level of the protein, progestagen-associated endometrial protein (PAEP). We show that PAEP gene over-expression promotes the proliferation, migration and invasion of tumor cells[3, 4], similar to the results presented by Uchida et al., the migration of human endometrial adenocarcinoma cells was stimulated by histone deacetylase inhibitors through the up-regulation of PAEP[5]. Although PAEP is a well-known lymphocyte regulatory molecule [6–8], its true function in the development and metastasis of melanoma in the tumor microenvironment remains unclear. This study aimed to analyze the role of PAEP immunomodulation in promoting tumor development.

PAEP, also known as glycodelin, is a secreted glycoprotein that was first isolated from human placenta, amniotic fluid, pregnancy deciduas and seminal plasma [9]. Many studies have shown that it can suppress the immune activity of T cells and play an important regulatory role in the establishment of maternal immune tolerance and human leukocyte antigen (HLA)-haploidentical embryo implantation[10, 11]. Considering the methodological similarities between tumor invasion and embryo implantation[12, 13], we hypothesized that the suppressive activity of melanoma-derived PAEP protein on T cells in the tumor microenvironment may resemble that of endometria-derived PAEP protein on T cells during pregnancy.

## Materials and Methods

### Cells

This study was conducted according to the ethical guidelines of the 1975 Declaration of Helsinki, and approved by the ethics committee of Beijing Institute of Transfusion Medicine. All participations had given written informed consent and received considered protection. No monetary compensations were offered.

Metastatic melanoma cell line 624.38-Mel (HLA-A*0201/0301, B*1402/0702), a clone of melanoma cell line 624, was kindly presented by the National Cancer Institute, Surgery Branch (National Institutes of Health, Bethesda, MD, USA)[14] and maintained in RPMI-1640 culture media (Invitrogen, CA, USA) supplemented with 10% fetal bovine serum (FBS, Invitrogen). Peripheral blood lymphocytes (PBLs) were obtained, following Ficoll density gradient centrifugation (Propbs Biotechnology, Beijing, China), from the whole blood of normal healthy donors. CD3^+^, CD4^+^ and CD8^+^ T cells were enriched by MACS (Miltenyi Biotech, Bergisch Gladbach, Germany) according to the manufacturer’s protocol. Th1 cells (CD4^+^ CXCR3^+^) and Th1-free T helper cells (CD4^+^ CXCR3^-^) were isolated by Human Th1 cell isolation kit (Stemcell Technologies, Vancouver, Canada). Purity of the isolated cells was confirmed by direct staining with anti-CD3-FITC, anti-CD4-PE, anti-CD8-FITC (Miltenyi Biotech) or anti-CXCR3-eFluor 660 (eBioscience, CA, USA) followed by flow cytometric analysis. The numbers of CD4/CD8 cells and CD69/CD44 cells before and after activation were determined by direct staining with antibodies (Miltenyi Biotech) and flow cytometric analysis.

### Establishment of Stable PAEP knockdown cell lines

To obtain stable cell lines with a marked decrease of PAEP gene expression, 624.38-Mel melanoma cells were infected with PAEP shRNA (shPAEP) lentiviral particles 5’-ATAAACCCTTGGAGCATGA-3’. Non-targeting shRNA (shControl) lentiviral particles were applied as negative control. Following puromycin screening and expansion in culture, the stable transfectants were confirmed by Semi-quantitative Reverse Transcriptase Polymerase Chain Reaction (semi-qRT-PCR) and Western blotting analysis for mRNA and protein expression, respectively. The primers for RT-PCR were: PAEP-f-AAG TTG GCA GGG ACC TGG CAC TC; PAEP-r-ACG GCA CGG CTC TTC CAT CTG TT; β-actin-f-ACA CTG TGC CCA TCT ACG AGG; and β-actin-r-AGG GGC CGG ACT CGT CAT ACT, respectively.

### Preparation of melanoma-derived PAEP

PAEP was condensed from the serum-free culture supernatant of wild-type, shControl and shPAEP melanoma cells. First, a monolayer of melanoma cells at 80–90% confluence in serum-free RPMI1640 was incubated for 48 h. Culture supernatant was then collected and proteins (>10 kDa) were condensed using Vivaspin ultrafiltration spin columns (Sartorius, Goettingen, Germany) with a molecular weight cutoff of 10 kDa. The concentrations of total proteins with a molecular weight above 10 kDa and PAEP (26 kDa) in the condensed supernatant were determined by BCA protein assay (Thermo, NJ, USA) and PAEP ELISA kit (Uscn, Hubei, China), respectively.

### Cytokine assays

Purified CD4^+^ T helper cells, Th1 cells or Th1-free T helper cells were cultured in triplicate in 96-well plates at 1 × 10^5^ cells and 100 μl of RPMI-1640 medium supplemented with 10% FBS per well. T helper cells were activated with phytohemagglutinin (PHA) (10 μg/ml, Roche, Baden Württemberg, Germany) or coated anti-CD3 antibody (BD Biosciences, CA, USA) in the absence or presence of melanoma-derived PAEP. After 24 h, culture supernatants were collected. The levels of IL-2, IFN-γ, IL-4 and IL-5 in the medium were determined by ELISA (eBioscience) according to the manufacturer’s instructions. Data are expressed as the mean of triplicate samples. Comparable results were obtained in three separate experiments.

### Cell proliferation assay

PBLs were cultured in triplicate in 96-well plates at 4000 cells per well in RPMI-1640 medium supplemented with 10% FBS, 50 U/ml IL-2, 2.5 μg/ml CD3 monoclonal antibodies (eBioscience) and 2.5 μg/ml PHA. Melanoma-derived PAEP (condensed 624.38-Mel shControl supernatant) and PAEP-free 624.38-Mel shPAEP supernatant with the equivalent total quantity of melanoma-secreted proteins were added to these cells. Cell proliferation was assayed by MTS assay (Promega, WI, USA) for 4 consecutive days according to the manufacturer’s protocol. The solubilized formazan dye product was quantified spectrophotometrically at 490 nm with a 96-well plate reader.

### Apoptosis assay

Unfractionated PBLs and purified CD4^+^ and CD8^+^ T cells were individually activated with 50 U/ml IL-2, 2.5 μg/ml CD3 monoclonal antibodies and 2.5 μg/ml PHA for 36 h, with or without simultaneous treatment with the indicated concentrations of PAEP. Cells were then stained with annexin V-FITC and propidium iodide (PI) according to the manufacturer’s protocol. Data was acquired using the Cytomics FC 500 flow cytometer (Beckman Coulter 175487) and analyzed on CXP software (Beckman Coulter, CA, USA).

### HLA gene sequencing and HLA-A2 PCR amplification

HLA class I antigens of 624.38-Mel were typed by SeCore HLA Sequencing Kit (Life Technologies, CA, USA) and analyzed by uTYPE Software (Life Technologies) according to the manufacturer’s protocol. Since 624.38-Mel cells are HLA-A2 positive, to achieve effective antigen recognition, HLA-A2+ PBLs were screened by PCR amplification. The primers for HLA-A2 amplification were 5’-CCT CGT CCC AGG CTC T-3’ (sense) and 5’-TGG CCC CTG GTA CCC GT-3’ (antisense). The expected product was 813 bp. β-actin (396 bp) was used as internal standard.

### Cytotoxicity assay

The cytotoxicity of cytotoxic T lymphocytes (CTLs) against 624.38 cells was assessed by a lactate dehydrogenase (LDH) release assay, as previously described.[15] Briefly, 624.38-Mel shControl and 624.38-Mel shPAEP cells were irradiated with 30 Gy of ^60^Co gamma-rays and co-cultured with HLA-A2^+^ PBLs at a ratio of 1:5 for 4 days in RPMI-1640 medium supplemented with 10% FBS, 50 U/ml IL-2, 2.5 μg/ml CD3 monoclonal antibodies and 2.5 μg/ml PHA. The PBLs (effector cells) were then collected, counted by Trypan Blue staining and co-cultured with 4 × 10^3^ uninfected 624.38-Mel cells (target cells) at various ratios in 96-well U-bottom tissue culture plates for 4 h or 22 h. Cytotoxicity was assessed by an LDH assay of culture supernatant using the CytoTox 96 Non-Radioactive Cytotoxicity Assay kit (Promega) according to the manufacturer’s protocol. Data are expressed as mean ± standard deviation (SD) of triplicate wells and are representative of three experiments.

### Mixed lymphocyte tumor reaction

Proliferation of lymphocytes stimulated by transfected melanoma cells was detected by an MTS assay, as previously described[15]. Briefly, 624.38-Mel shControl and 624.38-Mel shPAEP cells were seeded into a 96-well plate. After inactivation with 30 Gy of ^60^Co gamma-ray irradiation, melanoma cells were co-cultured with HLA-A2^+^ PBLs at a stimulator-to-responder cell ratio of 1:5 for 4 days in RPMI-1640 medium supplemented with 10% FBS, 50 U/ml IL-2, 2.5 μg/ml CD3 monoclonal antibodies and 2.5 μg/ml PHA. Tumor cell and PBL spontaneous release controls were also setup. Then, MTS work solution was added to each well for the last 4 hours of the 4-day cultures, and the OD value at 490 nm was quantified spectrophotometrically with a 96 well plate reader. Finally, the percent of cytotoxicity was calculated using the following formula: %cytotoxicity = {1 - (experimental—PBL spontaneous) / tumor cell spontaneous} × 100.

### Statistical analysis

The ANOVA analysis for the multi-factor designs was applied and statistical differences between the experimental and control groups were assessed by Student’s *t*-test, utilizing SAS statistical software. The minimal level of significance was set at a value of p<0.05 with all data expressed as mean ± S.D.

## Results

### PAEP knockdown and preparation of melanoma-derived PAEP protein

**Fig 1:**
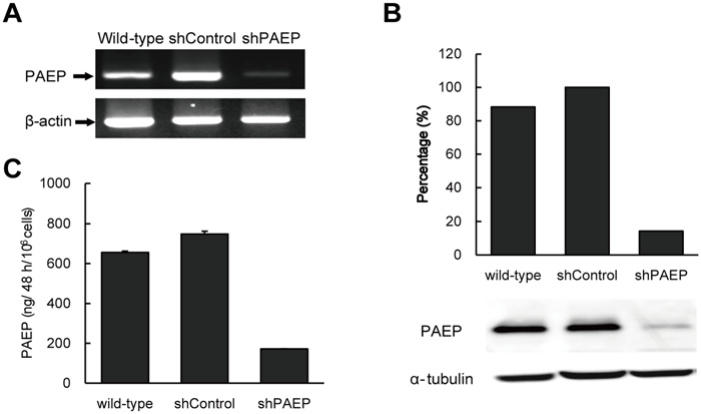
PAEP gene knockdown in melanoma cells. Stable PAEP 624.38-Mel shRNA transfectant and its non-targeting negative control transfectant, shControl, were established with a corresponding shRNA lentivirus. PAEP gene expressions were assayed by semi-quantitative RT-PCR (A) and Western blotting (B). PAEP protein secreted by melanoma cells was quantitated by ELISA (C).

PAEP is highly expressed and secreted by the majority of thick primary and metastatic melanoma cells. Two pairs of stable melanoma transfectants, 624.38-Mel shPAEP and 624.38-Mel shControl were established using PAEP lentiviral small hairpin RNA (shRNA) and non-targeting lentiviral shRNA, respectively. 624.38-Mel shPAEP showed an overall knockdown efficiency of over 80% both at the mRNA (Fig. 1A) and protein levels (Fig. 1B).

Melanoma-derived PAEP protein was prepared from the serum-free culture supernatant of PAEP-expressing 624.38-Mel shControl cells, while secreted proteins from PAEP-knockdown 624.38-Mel shPAEP cells were used as a negative control. Applying Vivaspin ultrafiltration spin columns with a molecular weight cutoff of 10 kDa, secretory proteins in serum-free culture supernatants, including PAEP of wild-type, shPAEP and shControl melanoma cells, were concentrated about 10-fold.

As shown in Fig. 1C, an ELISA assay indicates that the amount of PAEP protein produced by 10^6^ 624.38-Mel shControl cells in 48h was about the same as that of wild-type 624.38-Mel cells (748.2 ng and 655.8 ng, respectively). The PAEP protein in the 624.38-Mel shPAEP supernatant was much lower (172.0 ng), consistent with the results of semi-quantitative RT-PCR and Western blotting assays. The ratio of PAEP to total secreted proteins for shControl and shPAEP melanoma cells was about 1.6% and 0.3%, respectively.

### Inhibition of T helper cell activation by melanoma-derived PAEP

**Fig 2:**
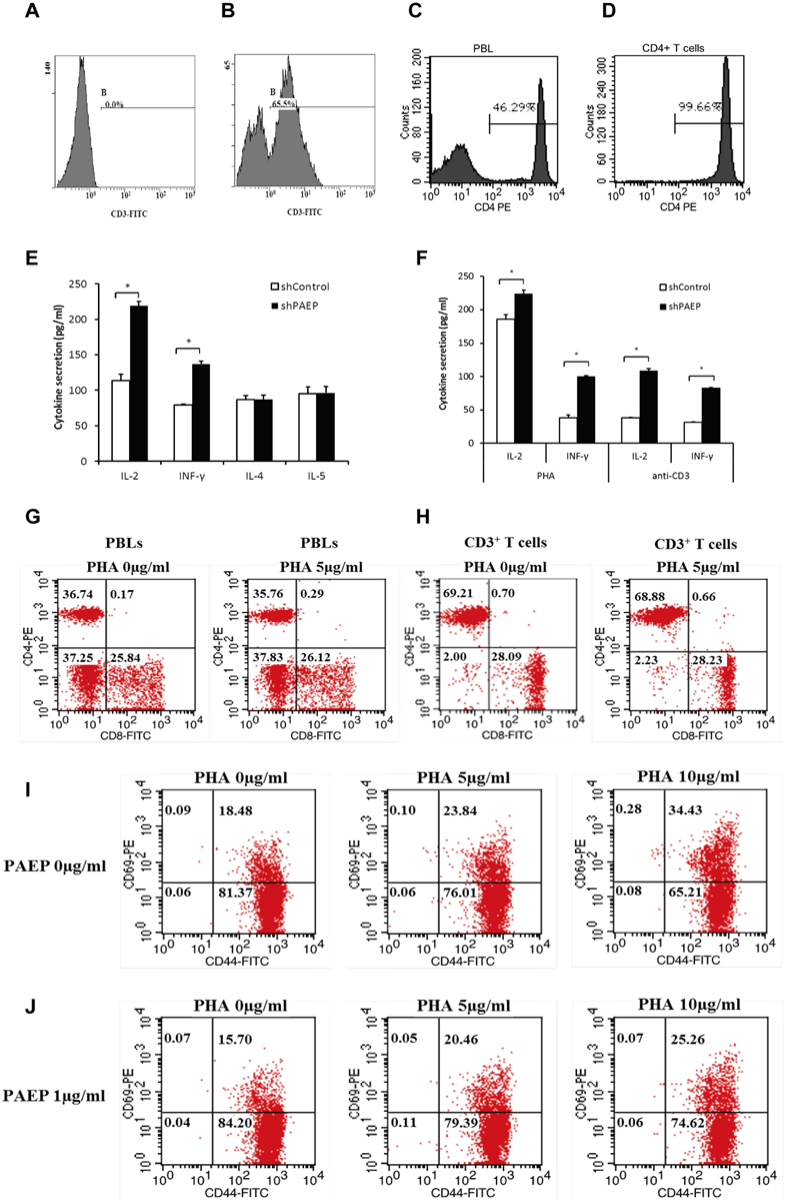
Activation of T helper lymphocytes by melanoma-derived PAEP protein. (A-B) The percent of CD3^+^ T cells in unfractionated PBLs from healthy donors was assayed for by direct staining with anti-CD3-FITC followed by flow cytometric analysis. (A) Negative control, (B) Healthy donor. Flow cytometry was applied to confirm the purity of CD4^+^ T cells before (C) and after (D) MACS isolation. (E) Levels of cytokines secreted by PHA-stimulated T helper cells co-cultured with PAEP-rich 624.38-Mel shControl or PAEP-poor 624.38-Mel shPAEP supernatant were determined. Melanoma-derived PAEP significantly inhibited both IL-2 and IFN- γ secretion by T helper cells (* p<0.05, Student’s *t*-test). (F) Levels of cytokines secreted by PHA- or anti-CD3 antibody-stimulated Th1 cells co-cultured with PAEP-rich 624.38-Mel shControl or PAEP-poor 624.38-Mel shPAEP supernatant were determined. Melanoma-derived PAEP significantly inhibited both IL-2 and IFN-γ secretion by Th1 cells (* p<0.05, Student’s *t*-test) (G-H) There were no differences in number of CD4/CD8 cells before or after PHA stimulation in the presence of PAEP (1 μg/ml). (G) PBLs; (H) CD3^+^ T cells. (I-J) The number of CD69/CD44 cells before and after activation in the absence or presence of PAEP was determined by direct staining with antibodies and flow cytometry. The number of CD69 cells significantly decreased co-cultured with PAEP-rich 624.38-Mel shControl supernatant. The experiment was repeated at least three times.

To examine the effect of melanoma-derived PAEP on the activation of T helper cells, CD4^+^ T cells were isolated from healthy donors. Flow cytometry analysis confirmed the percent of CD3^+^ T cells and the high purity (>99%) of enriched CD4^+^ T cells (Fig. 2A-D). T helper cells were activated with PHA in the absence or presence of melanoma-derived PAEP. Levels of Th1 (IL-2, IFN-γ) and Th2 (IL-4, IL-5) cytokines in conditioned medium during a 24-h cell culture were then determined. Melanoma-derived PAEP significantly inhibited the secretion of both IL-2 (113.8 ± 8.6 pg/ml, 624.38-Mel shControl group vs. 219.2 ± 6.3 pg/ml, 624.38-Mel shPAEP group, decreased by 48.1%, p<0.05) and IFN-γ (79.2 ± 1.3 pg/ml, 624.38-Mel shControl group vs. 137.2 ± 3.8 pg/ml, 624.38-Mel shPAEP group, decreased by 42.3%, p<0.05) but did not affect the PHA-stimulated secretion of IL-4 and IL-5 (Fig. 2E). Th1 (CD4^+^ CXCR3^+^) and Th1-free T helper cells (CD4^+^ CXCR3^-^) were further enriched from healthy donors and were activated with PHA or anti-CD3 antibody in the absence or presence of melanoma-derived PAEP. Melanoma-derived PAEP significantly inhibited the PHA-stimulated secretion of both IL-2 (decreased by 17.1%, p<0.05) and IFN-γ (decreased by 61.7%, p<0.05) from Th1 cells (Fig. 2F), but did not affect those secretions of Th1-free T helper cells. Similar results were obtained in CD3 antibody-stimulated Th1 or Th1-free helper cells (Fig. 2F). The results indicated that Melanoma-derived PAEP significantly inhibited the secretion of both IL-2 and IFN-γ from Th1 cells.

Moreover, in the presence of PAEP, there was no effect on the number of CD4/CD8 cells from PBLs (Fig. 2G) or CD3^+^ T cells (Fig. 2H) before or after activation. The number of CD69 cells significantly increased after PHA-stimulation and decreased in the presence of PAEP, whereas the number of CD44 cells did not change in the presence of absence of PAEP (Fig. 2I-J). Collectively, the results indicated that the PAEP protein secreted by melanoma cells primarily inhibited the activation of Th1 cells, but not Th2 cells. Since secretion of IL-2 is pivotal to the lymphocyte proliferative response and IFN-γ plays a key role in T cell cytotoxicity [16], the influence of melanoma-derived PAEP on the proliferation and cytotoxicity of T cells was then examined.

### Inhibition of PBL proliferation by tumor PAEP

**Fig 3:**
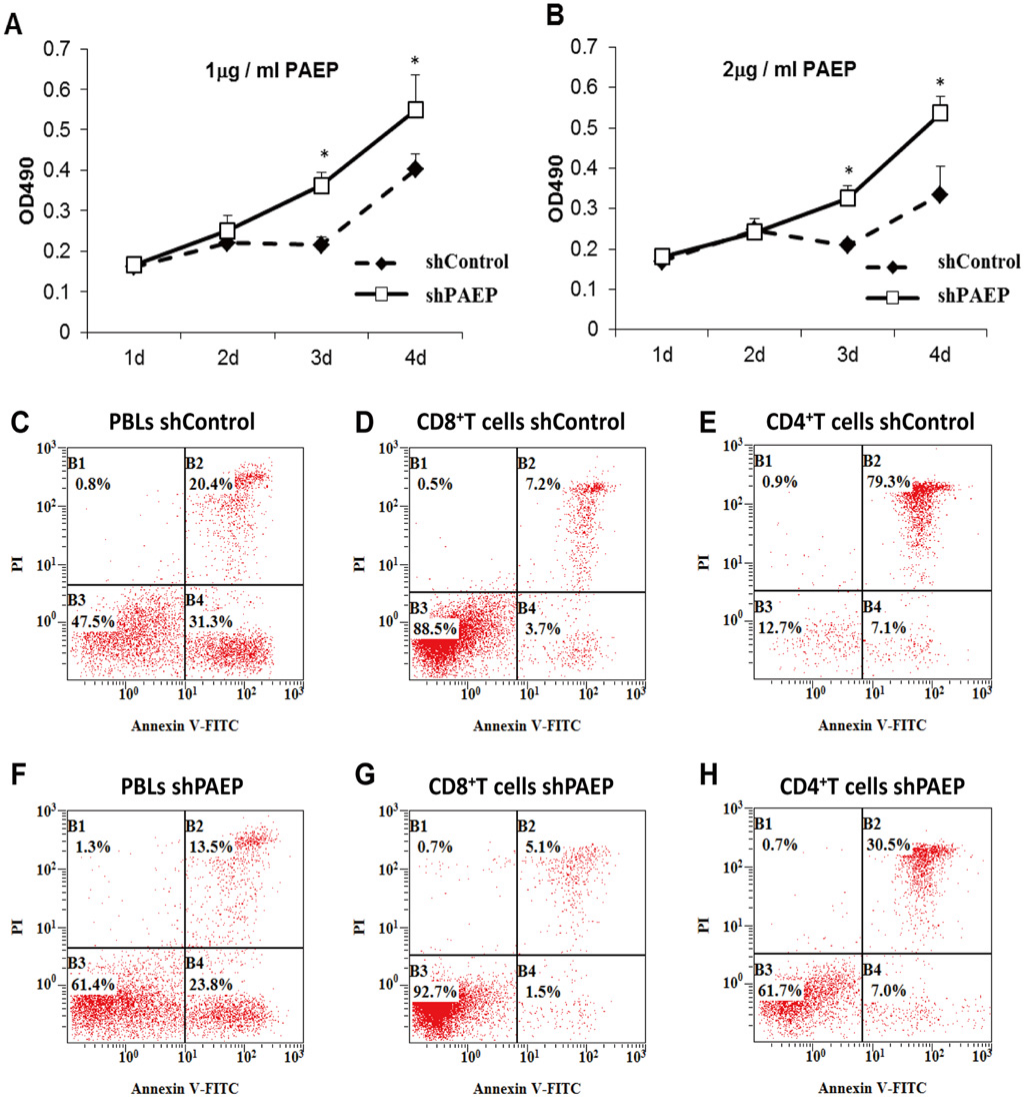
Proliferation and Apoptosis of lymphocyte affected by melanoma-derived PAEP protein. There was a significant difference of lymphocyte proliferation between PAEP-rich 624.38-Mel shControl group and PAEP-poor 624.38-Mel shPAEP group at concentrations of 1 μg/ml PAEP (A) and 2 μg/ml PAEP (B), indicating that proliferation of PBLs was significantly inhibited by PAEP (* P < 0.05, Student’s *t*-test). The early and late phase apoptosis of PBLs co-cultured with PAEP-rich 624.38-Mel shControl supernatant (equivalent to 1 μg/ml PAEP protein) for 36 h (C) was significantly increased compared with PBLs co-cultured with shPAEP supernatant (F). Similar results were obtained with purified CD8^+^ or CD4^+^ T cells (D vs. G, E vs. H). Results are presented from one representative experiment. The same experiment was repeated at least three times.

To determine the effect of melanoma-derived PAEP on activated PBLs, conditioned-media from melanoma cells expressing PAEP was added to PHA-simulated PBLs and viable cells measured by the CellTiter 96 Aqueous non-radioactive cell proliferation assay kit [15]. As shown in Fig. 3A-B, proliferation of PHA-stimulated PBLs was significantly inhibited by condensed serum-free culture supernatant from 624.38-Mel shControl cells, corresponding to estimated PAEP concentrations of 1 μg/ml (Fig. 3A) and 2 μg/ml (Fig. 3B), compared with that of the 624.38-Mel shPAEP group at days 3 and 4). This suggests that the melanoma-derived PAEP protein inhibit the proliferation of lymphocytes.

### Apoptosis of PBLs induced by tumor PAEP

Dual staining with Annexin V-FITC and PI was employed to determine whether PBL apoptosis was induced by melanoma-derived PAEP in unfractionated PBLs or purified CD8^+^ or CD4^+^ T cells. Following treatment with shControl supernatant (equivalent to 1 μg/ml PAEP protein) for 36 h, the early and late apoptosis of PBLs significantly increased compared with that of the shPAEP supernatant. Similar results were obtained with purified CD4^+^ and CD8^+^ T cells (Fig. 3C-H). These results indicate that PAEP-expressing melanoma cells are capable of inducing apoptosis of lymphocytes in the tumor microenvironment, with the observed proliferation inhibition of lymphocytes largely due to this increased apoptosis.

### Cytotoxicity of PBLs inhibited by tumor PAEP

**Fig 4:**
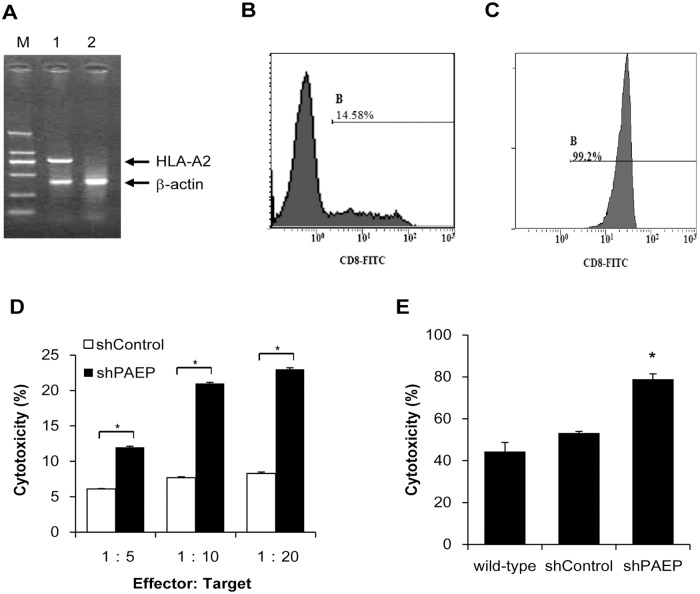
Cytotoxicity of CTLs inhibited by melanoma-derived PAEP. (A) HLA-A2^+^ PBLs were screened by DNA sequence-specific primer (SSP)-PCR. M: DL2000 marker (2000 bp, 1000 bp, 750 bp, 500 bp, 250 bp, 100 bp, from top to bottom). 1: HLA-A2 positive PBLs samples. 2: HLA-A2 negative PBLs samples. Flow cytometry was applied to confirm the purity of CD8^+^ T cells before (B) and after (C) MACS isolation. (D) Cytotoxicity of CTLs against target cells was assessed by colorimetric LDH assay and the data expressed as means ± SD of triplicate wells, representative of three experiments. HLA-A2+ PBLs primed with 624.38-Mel shPAEP cells showed more significantly increased cytotoxicity than those primed with 624.38-Mel shControl cells (* p<0.05, Student’s *t*-test) at effector-to-target ratios of 5:1, 10:1 and 20:1. (E) The cytotoxicity rate of CD8^+^ T lymphocytes co-cultured with 624.38-Mel shPAEP cells was much higher than those co-cultured with the 624.38-Mel shControl group. Results are shown for one of three independent experiments.

The effect of melanoma-derived PAEP on the cytotoxicity of T cells was also examined. HLA typing of melanoma cells was carried out by gene sequencing analysis prior to observing the interaction of individual lymphocytes and tumor cells. DNA sequencing showed that the 624.38-Mel cells were HLA-A2^+^. To efficiently present tumor antigens and elicit an immune response, PBLs from HLA-A2+ normal donors were identified as previously described [17] and then used for a cytotoxicity assay (Fig. 4A). CD8^+^ T cells were isolated by magnetically activated cell separation (MACS) for the mixed lymphocyte tumor reaction (MLTR). Flow cytometry analysis confirmed the high purity (>95%) of isolated CD8^+^ T cells (Fig. 4B-C).

In a LDH release assay (Fig. 4D), HLA-A2^+^ PBLs primed with PAEP-poor 624.38-Mel shPAEP cells showed significantly increased cytotoxicity than those primed with PAEP-rich 624.38-Mel shControl cells at effector-to-target ratios of 5:1, 10:1 and 20:1. This result was further confirmed by MLTR. Fig. 4E shows the results of one of three independent experiments. The cytotoxicity rate of lymphocytes co-cultured with PAEP-poor 624.38-Mel shPAEP cells was 78.9 ± 2.5%, much higher than that of the PAEP-rich 624.38-Mel shControl group (53.2 ± 0.8%) and the wild-type 624.38-Mel group (44.4 ± 4.3%). This suggests that the cytotoxicity of T cells is induced by PAEP-knockdown melanoma cells, showing that melanoma-derived PAEP protein inhibited the cytotoxicity of T cells.

## Discussion

Cellular immunity is an important part of the immune system. It plays a key role in the anti-tumor immune response, during which T cells are primarily involved in the killing of tumor cells and in controlling tumor cell growth [18, 19]. CD4^+^ T cells recognize peptides of exogenous antigens presented by MHC class II molecules and then activate CD8^+^ T cells and NK cells through the release of cytokines such as IL-2, IFN-γ and TNF, enhancing the cytotoxicity of effector cells and increasing the sensitivity of CTLs to target cells. CD8^+^ T cells recognize endogenous peptides presented by MHC class I molecules, differentiate into mature effector cells upon activation and subsequently lyse tumor cells with perforin or by inducing cell apoptosis [20].

As a defense, tumor cells secrete immunosuppressive factors and express certain proteins, such as TGF-β, IL-10 and VEGF, which negatively regulate the anti-tumor immune response and promote tumor growth [21–23]. The physiological role of one of these secreted proteins, PAEP, has been described as a direct T cell inhibitor [6] and has been shown to preferentially inhibit Th1 cytokine responses and chemokine expression when present during *ex vivo* priming of CD4^+^ T cells [8]. However, the precise role of PAEP in cancer remains uncertain.

PAEP is a glycoprotein of the lipocalin family, with four isoforms of PAEP identified according to their origin and carbohydrate structures [24]. PAEP isoforms with different glycosylation patterns show different functions, though they share an identical protein skeleton [24, 25]. The negatively charged sialic acid of glycodelin-A facilitates its contact with T cells [26], while the lack of sialic acid in glycodelin S prevents T cell access to its core apoptosis-inducing region and inhibits its functionality. Our previous study showed that PAEP derived from melanoma cells shares an identical protein skeleton with the four known isoforms [3]; however, its glycosylation pattern remains unclear. Considering the importance of glycosylation pattern in exerting protein functions and maximum extent mimic the tumor microenvironment in melanoma development, we applied concentrated supernatant as a source of melanoma-derived PAEP protein instead of recombinant PAEP or the purified protein, while that of PAEP-knockdown 624.38-Mel shPAEP cells were used as a negative control. Proteins smaller than 10 kDa, including most immunosuppressive and toxic factors, were discarded by an ultrafiltration spin columns with a molecular weight cutoff of 10 kDa.

PAEP has been suggested to contribute to maternal immune tolerance during embryogenesis by suppressing the activity of immune effector cells [7, 27–29]. In accordance with its physiological functions, PAEP has been shown to be frequently expressed in several gynecological malignancies [30–32]. We recently reported a high level of PAEP gene expression in melanoma, with a concomitant increase in the proliferation, migration and invasion of melanoma cells [3]. However, the true function and role of PAEP gene expression in tumor immune tolerance in the tumor microenvironment still is relatively unknown.

Considering the similarity between tumor invasion and embryonic implantation, we hypothesized that melanoma cell-secreted PAEP could inhibit lymphocyte activity and induce immune tolerance in the tumor microenvironment, promote tumor cell escape from immune surveillance and implantation and metastasis. Upon examination of the effects of melanoma-derived PAEP on the activation of purified CD4^+^ T helper cells, the secretion of IL-2 and IFN-γ, but not IL-4 and IL-5significantly decreased with the addition of PAEP-rich supernatant, indicating that the activation of Th1 cells was inhibited by melanoma-derived PAEP. PAEP-containing melanoma cell supernatant was then added to PBL culture conditions *in vitro*, and cell proliferation was observed for five consecutive days. Since a lower concentration of IL-2 induced by tumor PAEP might prevent lymphocytes from proliferation, an adequate exogenous IL-2 was added to each culture conditions. The cell growth curve showed that, compared with the control group, the proliferation of PBLs was significantly inhibited by the addition of melanoma-derived PAEP.

To explore the mechanism of PAEP-induced inhibition of T cell proliferation, unfractionated PBLs and purified CD8^+^ and CD4^+^ T cells were cultured with melanoma-derived PAEP. Early and late apoptotic ratios of lymphocytes were significantly increased, suggesting that the inhibition of PBL proliferation may be caused by the induction of apoptosis. Finally, we examined the effect of tumor PAEP on T cell-mediated tumor-specific cytotoxicity. HLA class I antigens play a key role in the anti-tumor immune response and are expressed on virtually all nucleated cells, while HLA-A2 is one of the most frequent HLA class I specificities and commonly used as a model to study HLA-A2-restricted CTL responses [33–35]. In case the T cells in shControl group were dying due to lack of IL-2, an adequate exogenous IL-2 was added to each culture conditions. The tumor-stimulated PBLs (effector cells) were counted by Trypan blue staining before they co-cultured with melanoma cells (target cells), insuring the accuracy of Effector: Target ratio. MLTR and CTL cytotoxicity experiments demonstrated that the cytotoxicity of HLA-A2^+^ CD8^+^ T cells significantly increased by silencing the PAEP gene of HLA-A2^+^ 624.38-Mel cells.

Since PAEP was first discovered in cancers of the reproductive system, cancer related PAEP research has been mainly confined to the reproductive system [30–32, 36]. In addition, studies on PAEP and tumor immunity are limited. Based on the fact that cytotoxicity mediated by T cells plays a key role in melanoma immunity, the immunosuppression of T cells induced by melanoma-derived PAEP protein has been explored in this study. Our results suggest that, similar to its role in embryo implantation, the synthesis and secretion of PAEP can be induced by tumor cells to inhibit T cell activation, proliferation and tumor-specific cytotoxicity in the tumor microenvironment. *In vivo* experiments are needed to further verify the suppressive effects of PAEP on lymphocyte function. PAEP, therefore, may be an important target in the search for effective immunotherapeutic strategies to control tumor growth in melanoma patients.
